# Sequencing improves our ability to study threatened migratory species: Genetic population assignment in California's Central Valley Chinook salmon

**DOI:** 10.1002/ece3.2493

**Published:** 2016-10-05

**Authors:** Mariah H. Meek, Melinda R. Baerwald, Molly R. Stephens, Alisha Goodbla, Michael R. Miller, Katharine M. H. Tomalty, Bernie May

**Affiliations:** ^1^ Department of Natural Resources Cornell University Ithaca NY USA; ^2^ Department of Animal Science University of California Davis Davis CA USA; ^3^ School of Natural Science University of California, Merced Merced CA USA

**Keywords:** Central Valley, fish, genetic stock identification, linkage map, management, RAD‐sequencing

## Abstract

Effective conservation and management of migratory species requires accurate identification of unique populations, even as they mix along their migratory corridors. While telemetry has historically been used to study migratory animal movement and habitat use patterns, genomic tools are emerging as a superior alternative in many ways, allowing large‐scale application at reduced costs. Here, we demonstrate the usefulness of genomic resources for identifying single‐nucleotide polymorphisms (SNPs) that allow fast and accurate identification of the imperiled Chinook salmon in the Great Central Valley of California. We show that 80 well‐chosen loci, drawn from a pool of over 11,500 SNPs developed from restriction site‐associated DNA sequencing, can accurately identify Chinook salmon runs and select populations within run. No other SNP panel for Central Valley Chinook salmon has been able to achieve the high accuracy of assignment we show here. This panel will greatly improve our ability to study and manage this ecologically, economically, and socially important species and demonstrates the great utility of using genomics to study migratory species.

## 1 Introduction

Animal migration is one of nature's most widespread occurrences, with migratory behavior present across all major branches of the animal kingdom (Alerstam, Hedenstrom, & Akesson, 2003; Dingle, 1996). Despite its ubiquity, migratory animals are decreasing in abundance worldwide and being listed as species of conservation concern at alarming rates (Wilcove & Wikelski, 2008). Protection of threatened migratory animals is often limited by an inability to identify individuals to their natal populations when multiple populations mix along the migratory pathway or experience population impacts at one or more points along these pathways (e.g., salmon, Larson, Seeb, Pascal, Templin, & Seeb, 2014; birds, Ruegg et al., 2014). Additional complexity is created when management goals seek to maintain populations for long‐term persistence, as well as manage populations for human consumption and/or recreational activities (i.e., fishing and hunting). Discrete management of populations minimizes the potential for undetected extinction of unique lineages and preserves diversity within the population complex (Hilborn, Quinn, Schindler, & Rogers, 2003; Schindler et al., 2010). Accurate population assignment also allows managers to determine where specific populations are most negatively affected along their migratory corridors and to evaluate the effect of local stressors on population declines (Marra, Hobson, & Holmes, 1998; Norris & Taylor, 2006).

Accurately identifying distinct populations, the individuals that belong to those populations, and their habitat use patterns during migration requires tools that match the scale and precision desired for a given question. Telemetry has historically been used to study the movement of migratory animals (e.g., Bonfil et al., 2005; Croxall, Silk, Phillips, Afanasyev, & Briggs, 2005), but is impractical for large‐scale applications, often not feasible for small individuals, and can be overly invasive for species of conservation concern (Adams, Rondorf, Evans, & Kelly, 1998; Hebblewhite & Haydon, 2010; Jepsen, Koed, Thorstad, & Baras, 2002). Genetic assignment, however, can provide a more reliable method for tracking individuals and movement across life‐history stages and can be accomplished at large scales. New advances in genome sequencing provide an opportunity to scan thousands of markers to identify a select subset needed for accurate population assignment (Amish et al., 2012; Hess et al., 2014; Lao, Duijn, Kersbergen, de Knijff, & Kayser, 2006). This approach expands our ability to track the movements, population structure, habitat use, and impacts on a large numbers of individuals from migratory populations, while minimizing handling and sampling stress (Davey et al., 2011).

Chinook salmon (*Oncorhynchus tshawytscha,* Walbaum 1792, Figure 1) are an important migratory keystone species and provide great economic and social value via recreational, commercial, and heritage fisheries (Cone, 1995; Lichatowich, 1999; Williams, 2006). They also typify the necessity for identifying populations and the individuals that belong to them to facilitate protection and management. Chinook salmon are anadromous—eggs are laid and hatch in freshwater where juveniles rear before migrating to the ocean to grow and mature, after which they typically migrate back to their natal freshwater streams to spawn and die. Spatial and temporal variation has led to the evolution of distinct “runs” that each take advantage of unique environmental conditions. Runs are named for the timing of their spawning migration (e.g., Fall run, Spring run), and a single river system can support multiple unique Chinook runs. The runs mix as adults and juveniles, sharing common migratory pathways during both spawning and seaward migrations, as well as sharing juvenile rearing habitat. There are no obvious morphological characteristics that identify an individual's run (del Rosario et al., 2013; Williams, 2006), but management goals are mandated for each run separately (Good, Waples, & Adams, 2005).

**Figure 1:**
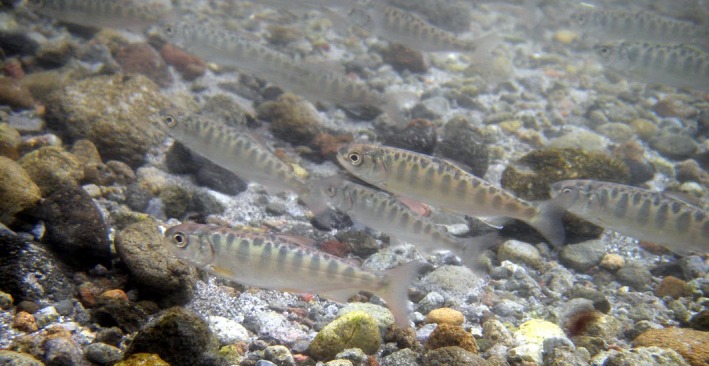
Juvenile Chinook salmon. Photograph credit: Carson Jeffres

The Great Central Valley of California includes the southernmost edge of the Chinook salmon species range and supports the most diverse assemblage of life‐history types anywhere, including four runs: Spring, Fall, Late fall, and the endemic Winter run (Fisher, 1994; Yoshiyama, Fisher, & Moyle, 1998). The Central Valley was once one of the most productive U.S. Pacific salmon systems, yet now all four Chinook salmon runs have declined to a fraction of their historical abundance (Yoshiyama et al., 1998), with Spring and Winter runs being listed as threatened and endangered, respectively, under the federal Endangered Species Act and Fall run and Late fall as species of concern (NOAA‐NMFS 1999, 2005). Each run is managed as a separate evolutionary significant unit (ESU), warranting species level protection, with the exception of Fall and Late‐fall run, which are listed as a single ESU. Differentiating between runs is critical for limiting negative impacts to the ESUs, identifying and quantifying mortality, monitoring populations, targeting restoration efforts, and managing the fishery. Yet, despite efforts to target management, monitoring, and conservation to run, there has been somewhat limited success using genetic or morphometric methods in identifying Central Valley Chinook back to their source runs and populations (Harvey, Jacobson, & Banks, 2014).

The primary method currently used to determine the run of Chinook salmon as they migrate through the Central Valley is phenotypic—it is based on size class projections over time and termed the “length‐at‐date” criteria (del Rosario et al., 2013; Harvey & Stroble, 2013; —for detailed explanation of the criteria see Harvey et al., 2014). Unfortunately, this method of run assignment has been shown to be very inaccurate, with over 50% of the individuals classified as Winter run belonging to Spring, Fall, or Late‐fall runs (Harvey et al., 2014).

Genetic resources to differentiate Central Valley Chinook salmon populations thus far have been limited, with Winter run being the only run that can reliably be distinguished (reviewed in Lindley et al., 2004). Microsatellite markers have shed light on population genetic structure among runs (Banks, Rashbrook, Calavetta, Dean, & Hedgecock, 2000; Garza, Blankenship, Lemaire, & Charrier, 2008; Hedgecock, 2002; Nielsen, Pavey, Wiacek, & Williams, 2005; Williamson & May, 2005), but have been insufficient for providing genetic assignment between populations characterized with low genetic divergence, such as Fall and Late‐fall runs and among Spring run populations. Additionally, microsatellites are difficult to implement in a high‐throughput manner (Hauser, Baird, Hilborn, Seeb, & Seeb, 2011; Smith, Seeb, Schwenke, & Seeb, 2005), require extensive effort to standardize among laboratories (Seeb, Antonovich, Banks, & Beacham, 2007), and can be fraught with technical problems (Narum et al., 2008). Single‐nucleotide polymorphisms (SNPs) are becoming the marker of choice, given their density throughout the genome, ease of detection via next‐generation sequencing, high‐throughput capabilities, and reproducibility across molecular laboratories. SNP markers were recently developed (Clemento, Abadía‐Cardoso, Starks, & Garza, 2011) in Chinook salmon for high‐throughput analysis; however, this panel of 96 SNP markers was designed to distinguish Chinook salmon from different regions along the West Coast and does not adequately discriminate all runs within the Central Valley, nor does it distinguish among the imperiled Spring run populations (Clemento, Crandall, Garza, & Anderson, 2014; Meek et al., 2014). Due to the threatened status of Spring run, it is important to be able to manage genetically distinct populations within the run to protect diversity within the population complex (Carlson & Satterthwaite, 2011).

We developed a high‐resolution SNP assay panel to identify and study Chinook salmon in the Central Valley. Using restriction site‐associated DNA sequencing (RADseq, Miller, Dunham, Amores, Cresko, & Johnson, 2007; Baird et al., 2008), we scanned the genomes of Chinook salmon sampled from across the Central Valley and identified thousands of new SNPs distributed across the genome. We then developed a genetic linkage map and a small subset of ancestry‐informative markers (AIMs; Rosenberg, Li, Ward, & Pritchard, 2003) for differentiating Chinook salmon run timing via Fluidigm SNP Type assays, to allow for high‐throughput and rapid run identification and identification of unique populations within Spring run. Genetic resources that rapidly and reliably distinguish runs and populations of Central Valley Chinook will be immensely valuable for genetic management and monitoring, as well as studies of life‐history trait evolution, genomewide association, habitat use, and other questions of ecological or evolutionary interest in this threatened migratory species.

## 2 Materials and Methods

### 2.1 Sample collection

**Figure 2:**
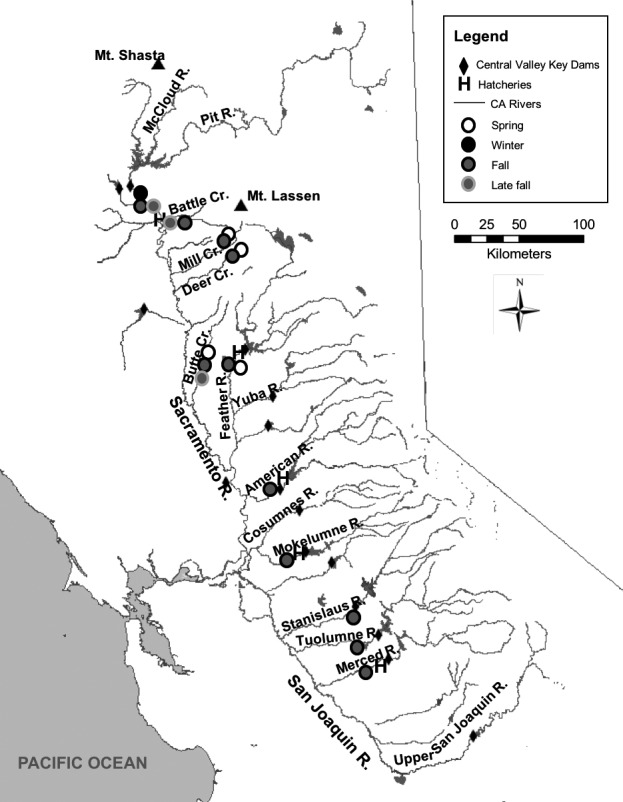
Populations sampled. Locations of dots do not represent exact location of samples collected, but rather the existence of a particular run in a given river

We obtained adult Chinook salmon fin clip tissues from the California Dept. of Fish and Wildlife Anadromous Resources Tissue Archive. Individuals were collected during spawning migrations, from all major tributaries in the Central Valley with known spawning populations for all four Chinook salmon runs across multiple years (Table 1, Figure 2).

**Table 1:** Sample numbers from each population of interest and location abbreviations. The AIM panel column shows the number of individuals used in the blind assignment test of the AIM assay SNP panel (the “holdout” set). The number of samples used in the blind assignment test are those that genotyped at >70% of the Fluidigm AIM panel loci

Location	Years sampled	Location abbreviation	RADseq sample size	AIM panel sample size
Fall Run			63	68
Battle Cr.	2002	F_BTC	2	–
Butte Cr.	2002–2004	F_BUT	10	6
Deer Cr.	2002–2004	F_DER	10	4
Feather R. Fish Hatchery	2007–2011	F_FRH	–	30
Merced R.	2008	F_MER	10	–
Mill Cr.	2002–2004	F_MIL	9	4
Mokelumne R. Fish Hatchery	2005	F_MKH	2	–
Merced R. Fish Hatchery	2001–2004	F_MRH	17	5
Nimbus Fish Hatchery	2002–2005	F_NIM	–	6
Stanislaus R.	2001, 2002, 2008	F_STN	–	6
Tuolumne R.	2001, 2003, 2004, 2008	F_TOU	10	7
Upper Sacramento R.	2002	F_USR	2	–
Late Fall Run			36	75
Battle Cr.	2003	L_BTC	2	–
Butte Cr.	2000	L_BUT	2	–
Coleman National Fish Hatchery	1998, 1996, 2000	L_COL	2	40
Upper Sacramento R.	2003, 2004, 2005	L_USR	30	35
Spring Run			93	137
Butte Cr.	2004, 2006–2009	S_BUT	30	37
Deer Cr.	2002, 2003, 2005	S_DER	31	35
Mill Cr.	2000–2002, 2004, 2005	S_MIL	32	37
Feather R. Fish Hatchery	2006–2010	S_FRH	–	28
Winter Run			30	40
Upper Sacramento R.	2001, 2002	W_USR	30	40

### 2.2 Molecular biology

We constructed RAD libraries using individuals from all the major tributaries in the Central Valley that have consistent Chinook spawning populations and the main stem of the Sacramento River (Table 1; Figure 2). Feather River Hatchery samples were sequenced but not included in our AIM panel selection because past hatchery practices have led to introgression between the Fall and Spring run hatchery populations (California HSRG, 2012; Garza et al., 2008). We extracted genomic DNA from all samples using the Qiagen DNeasy 96 Blood and Tissue extraction kit, quantified DNA concentrations using the Invitrogen Qubit Assay Kit (Life Technologies, Carlsbad, California), and normalized all samples to 25 ng/μl. RAD libraries were constructed using the *SbfI* restriction enzyme following the protocol described by Lew et al. (2015). We ligated each sample with a unique custom six base pair barcode and then multiplexed 30–47 individuals per library. Libraries were sequenced via eight lanes of 100 base pair single‐end reads and one lane of 100 base pair paired‐end reads on an Illumina HiSeq 2000 (Vincent J. Coates Genomics Sequencing Laboratory, Berkeley, CA). This included one library of individuals that were resequenced due to low read coverage (fewer than 1,000,000 sequences) in the first sequencing run.

### 2.3 SNP discovery and genotyping

We performed SNP discovery following the bioinformatics methods detailed in Miller et al. (2012). One hundred base pair reads were trimmed from the 3′ end to 92 bp, and reads were filtered to eliminate those with a >20% probability of sequencing error, or those that contained one or more ambiguous base calls. The six base sample‐specific barcode and the partial *SbfI* site sequence were also removed from the sequence.

We conducted three rounds of SNP discovery, using a different ascertainment panel in each. To discover SNPs that are polymorphic across Chinook salmon runs, we used four individuals, taken from across multiple locations, from each run type (Fall: TOU, MIL, MKH, BTC; Late fall: COL, BUT, USR; Winter: USR; Spring: BUT, MIL, DER) in the ascertainment panel (Table 1). We looked for SNPs within Fall and Spring runs with two additional rounds of SNP discovery. For the Spring run discovery, we used two individuals each from the extant wild populations: Mill, Deer, and Butte creeks. For the Fall run discovery, we used two individuals from each of four locations, which represent populations from both the San Joaquin River drainage and the Sacramento River drainage: Mill, Butte, Tuolumne, and Merced Rivers. We did not conduct separate discoveries for Winter and Late‐fall runs. The number of reads per individual after quality filtering ranged between ~1.8 and 10 million. For those over 3 million, we subsampled each individual to 3 million reads. We identified SNPs using the program Novoalign (Novocraft Technologies). We ran Novoalign in exhaustive mode, showing 150 randomly selected alignments per read, and an alignment score threshold of 120. Using the output of this alignment, we then filtered based on the following criteria: Only loci with a single SNP were retained, sequences with less than two external alignments were ignored, only alignments with two alleles were retained, the minimum samples per allele was one, the minimum sample count per locus was three, and the maximum Novoalign alignment score between any sequences in a locus was 30.

We combined the identified loci across all three discoveries, removing duplicate loci, and then genotyped all 222 sequenced individuals at all the identified loci. We used the program Bowtie (Langmead, Trapnell, Pop, & Salzberg, 2009) to index this file and align the quality‐filtered reads from all individuals, allowing only one mismatch in the alignment. We counted the number of reads with a perfect match to each allele for each individual. If the sum of the total reads for a locus was less than six for an individual, we scored the genotype as unknown. To convert read counts to genotypes, we took the log ratio of the number of reads of allele 1 to number of reads of allele 2 at each locus (Lew et al., 2015). We then excluded those that only had counts for one allele (obvious homozygotes) and calculated the mean and standard deviation of the remaining log ratio distribution. We called everything within 1.5 standard deviations from zero a heterozygote. Homozygotes were called when 0.85 or greater proportion of the reads were for a single allele. Everything that fell between the homozygote and heterozygote ranges was recorded as unknown.

### 2.4 Quality filtering

We filtered the dataset to remove low‐coverage SNPs and potential paralogs. We removed loci that were not genotyped in at least ten individuals from each run and excluded individuals that were genotyped at an unusually low number of loci (less than two standard deviations below the mean number of genotyped loci per individual). We then filtered out loci that were typed at <70% of the remaining individuals. We removed loci that had a minor allele frequency of <0.01. We also removed loci with observed heterozygosity >0.55, given that the expectation for biallelic SNPs is a maximum observed heterozygosity of 0.5. We removed all the SNPs located in the last two base pair positions of a sequence, as there were considerably more SNPs in these positions than the other base pair positions. This is likely driven by the fact that Illumina sequencing is more error prone toward the sequence terminal positions (Minoche, Dohm, & Himmelbauer, 2011).

### 2.5 Linkage mapping

We created a linkage map to evaluate the location of discovered SNPs across the genome using JoinMap 4.0 (Van Ooijen, 2006). We sequenced and genotyped individuals from three full‐sib Fall run families (48 progeny/family; Williamson et al. 2008) using the above methods. Loci were removed that had: (1) missing parental genotype data or >20% missing data in the progeny or (2) evidence of duplication due to deviation from expected Mendelian segregation ratios. Linkage groups were formed using a minimum LOD score of 7.0. Within linkage groups, loci were ordered using regression mapping with a recombination frequency threshold of 0.4, a LOD threshold of 1.0 and a jump threshold of 5.0. A ripple was performed after each locus addition, and distance was calculated using Kosambi's mapping function. We independently created linkage groups for each family and then combined them into a consensus map. Linkage groups were assigned to Chinook salmon chromosomes (Phillips, Park, & Naish, 2013) by mapping loci to previously mapped SNP loci (Brieuc, Waters, Seeb, & Naish, 2014; McKinney et al., 2016) with the NCBI MegaBLAST algorithm (Zhang, Schwartz, Wagner, & Miller, 2000) using default parameters. A positive match occurred if sequence similarity was >98% (i.e., ≤1 mismatch, corresponding to SNP location).

### 2.6 AIM selection and assay development

To choose the best loci for the AIM panel, we calculated the pairwise allele frequency difference for each locus among all the different runs, and locations within Spring run (DER, MIL, BUT). Prior to calculating this, we removed ten randomly chosen individuals from each group and set them aside to be used in the initial testing of assignment accuracy. We determined the AIM panel by first choosing the top twenty loci that had the highest allele frequency differences for each pairwise comparison. We also ranked loci based on F_ST_ and informativeness (I_n_) (Rosenberg et al., 2003), but found they did similarly (data not shown), so proceeded with the allele frequency difference ranking, as this ranking resulted in slightly higher assignment accuracies. We tested for linkage disequilibria in all pairwise comparisons within each run and locations within Spring run using the program GENEPOP (Raymond & Rousset, 1995; Rousset, 2008). We removed loci from the AIM panel that were out of linkage equilibrium in more than one comparison. We also evaluated marker location on the Chinook linkage map to ensure no loci that we chose for the panel were tightly linked and to ensure coverage across linkage groups. We obtained sequence reads long enough to allow assay design by aligning the forward and reverse reads of the paired‐end library. Using custom Perl scripts (available upon request), we required 30 bases of identical overlap between the end of the forward read and the beginning of the reverse read to ensure accurate alignment. If a longer sequence for a particular locus could not be constructed, or if the alignment of the forward and reverse reads was ambiguous (with a forward or reverse read aligning to more than one locus), the locus was excluded. We also removed any loci where the SNP was too close to the sequence end for primer design, as well as any loci that did not meet Fluidigm's design standards. Anytime a locus was removed from the AIM panel for any of the above reason, we replaced it with the next highest ranked locus for the comparison for which it was informative. We developed candidate loci into Fluidigm SNP Type assays using Fluidigm's D3 Assay Design system. Prior to ordering the assays, we tested assignment accuracy of the final AIM panel by assigning the subset of individuals we had removed from the RADseq dataset (prior to calculating allele frequencies) back to the remaining RADseq dataset (data not shown).

### 2.7 Validation of AIM panel

We validated the Fluidigm SNP Type assays by following the manufacturer's protocols for genotyping. We first genotyped 32 individuals sampled from across the different sampling locations that had been included in sequencing effort, to ensure the genotypes determined by the assays were congruent with our RADseq genotypes. We removed any loci that had more than two mismatches in genotypes, resulting in a panel with <5% mismatches in allele calls between the two methods. We then employed a “training‐holdout” procedure to test AIM panel accuracy by evaluating the performance of the AIM panel when assigning additional individuals (the “holdout” set), which were not used in the “training set” to rank SNPs, thus avoiding high‐grading bias (Anderson, 2010). We genotyped at least 40 additional individuals per run and per Spring run location (Table 1) for the “holdout” set, using the SNP Type assays. This dataset also included Spring and Fall run individuals from the Feather River Hatchery, to assess how well our AIM panel could distinguish the Spring run from the Fall run fish in this hatchery, despite the past hatchery practices that have lead to introgression. We removed any individuals from the test that were not genotyped in at least 70% of the loci. We assigned individuals back to run using the full, filtered RADseq dataset as the baseline and the program ONCOR (Kalinowski, Manlove, & Taper, 2008). We removed 23 individuals from the baseline whose run was clearly misidentified at the time of sampling, based on structure analysis using the full SNP dataset (data not shown). We tested assignment accuracies for Spring run samples by assigning them to the individual tributaries, to Butte and Mill/Deer Creek reporting groups, as well as to a larger Spring run reporting group. An 0.80–0.90 assignment probability cutoff is commonly used to assign individuals to populations (e.g., Clemento et al., 2014; Daly et al., 2012), yet the higher the probability of assignment required, the fewer samples can be assigned. To investigate the effects of using different assignment probability cutoffs, we used cutoffs of both 0.08 and 0.06 probabilities of assignment. Assignment accuracy was calculated as the proportion of correct assignments to the total number of assignments made per group, excluding those that could not be assigned due to low assignment probabilities. To characterize the effect of low assignment probabilities on the performance of the AIM panel, we also calculated the proportion of the samples that could not be assigned due to low assignment probabilities, allowing future users to weigh the trade‐offs between using different assignment probability cutoffs. Additionally, we evaluated the utility of the AIM panel for conducting mixed stock analyses—a scenario that is relevant for ocean fisheries to determine what proportion of a fishery catch belongs to each reporting group. To do this, we implemented the realistic fishery simulation in ONCOR. We ran 1,000 mixture simulations of 200 individuals per mixture. Table 2 shows the proportions of each group used in the mixture simulation.

**Table 2:** Results of the realistic fishery simulation in ONCOR

Reporting group	Simulated proportion	Estimated proportion	Standard deviation	95% Confidence interval
Fall	0.25	0.2712	0.0334	(0.2043, 0.3396)
Late Fall	0.25	0.2309	0.0324	(0.1677, 0.2967)
Spring‐Mill/Deer	0.15	0.1480	0.0248	(0.1001, 0.1972)
Spring‐Butte	0.10	0.1002	0.0210	(0.0603, 0.1412)
Winter	0.25	0.2497	0.0302	(0.1950, 0.3100)

## 3 Results

### 3.1 SNP discovery and genotyping

The sequencing effort resulted in an average of 232,000,898 sequence reads per library (range: 136,417,117–460,215,599) and 4,072,681 reads per individual (range: 209,815–24,137,958).

The run comparison discovery resulted in 15,854 polymorphic loci, the Fall run discovery resulted in 14,670 loci, and the Spring run discovery resulted in 14,466 loci. After combining the datasets and removing duplicate loci across discoveries, the final discovery had 24,198 unique SNP loci. The mean number of loci that each individual was genotyped at was 18,041 (95% CI: 6,621–22,969). We removed six individuals that were typed at fewer than the lower confidence interval (<6,621 loci). After removing loci for failing to meet quality‐filtering standards, our final SNP dataset contained 11,783 biallelic SNPs.

### 3.2 Linkage mapping

A linkage map containing 34 linkage groups was constructed using a total of 4,600 SNP loci. We were able to align 2,560 of our loci to previously mapped loci. The total length of the linkage map was 1,815.8 cM, with individual linkage group distances ranging from 28.6 cM (Ots29) to 83.6 cM (Ots14). The average distance between loci was 0.40 cM. The number of loci per linkage group ranged from 37 (Ots24, Ots32) to 303 (Ots01), with an average of 135 loci per group (see Table S1).

### 3.3 AIM selection

Based on the allele frequency difference rankings and the linkage map, we developed a suite of 114 Fluidigm SNP Type assays for the AIM panel. We excluded 34 of these assays from the final AIM panel because they either failed to properly amplify as assays or the genotypes did not meet matching criteria between the sequencing and assay efforts. All mismatches were heterozygote versus homozygote mismatches, so most likely allelic dropout in one of the genotypes (with the RADseq data producing the homozygote and the SNP Type assay producing the heterozygote most often). Therefore, our final AIM assay panel contained 80 SNP assays. These markers covered at least 25 of the 34 chromosomes, with several loci spread across each linkage group (see Table S2).

### 3.4 Validation of AIM panel

The validation of the AIM panel showed high accuracy of assigning individuals to their run types (Table 3). When using the 0.80 probability of assignment requirement, assignment accuracies were >90% for Fall run and averaged 76% for Late fall. Spring run populations assigned to a Spring run reporting group with an average of 96% assignment accuracy. Assignment accuracy of Winter run was 100%. The distinction between Mill and Deer Creek Spring run was not strong enough to get useful assignment accuracies to specific tributaries (33%–54%). However, collapsing Mill and Deer down to a single Mill/Deer reporting group allowed assignment accuracy of 77%. The majority of both the Fall and Spring run from the Feather River Hatchery assigned to Fall run (≥74% and 70%, respectively). We also tested whether the Spring run from the Feather River Hatchery could be assigned to its own reporting group. This, however, achieved very low assignment accuracy (data not shown).

**Table 3:** Assignment accuracies with the AIM panel, using 0.80 and 0.60 assignment probability cutoffs. The first column is the putative run (as identified at time of sampling), with number of samples assigned in parentheses. The columns are the proportion of those samples that were assigned to the different groups. The last column displays the percent of the total number genotyped that could be assigned at the given threshold. Shaded boxes highlight the putative correct assignments. S_FRH is not shaded because past hatchery practices have lead to introgression between Fall and Spring run in the hatchery population, making the “correct” assignment unclear. See Table 1 for location acronyms. “Fall” includes all sampled Fall run locations except FRH. A) Assignment accuracy of AIM panel using Run as the reporting group, B) assignment accuracy of AIM panel splitting Spring run into Butte Cr. and Mill/Deer creek reporting groups

Putative run	Fall	Late fall	Spring	Winter	% of total assigned
A) Run reporting group					
Assigned at 0.80 probability					
Fall (32)	0.91	0.09	0	0	63
F‐FRH (19)	0.74	0.11	0.16	0	84
L_COL (26)	0.23	0.77	0	0	65
L_USR (28)	0.25	0.75	0	0	80
S_MIL (33)	0.06	0	0.94	0	89
S_DER (34)	0.03	0	0.97	0	97
S_BUT (36)	0	0	0.97	0.03	97
S_FRH (22)	0.73	0.09	0.18	0	79
Winter (40)	0	0	0	1.00	100
Assigned at 0.60 probability					
Fall (34)	0.85	0.15	0	0	89
F‐FRH (27)	0.81	0.07	0.11	0	90
L_COL (33)	0.27	0.73	0	0	82
L_USR (34)	0.26	0.74	0	0	97
S_MIL (34)	0.06	0	0.94	0	92
S_DER (35)	0.03	0	0.97	0	100
S_BUT (37)	0	0	0.97	0.03	100
S_FRH (26)	0.73	0.08	0.19	0	93
Winter (40)	0	0	0	1.00	100

Putative run	Fall	Late fall	Spring‐Mill/Deer	Spring‐Butter	Winter	% of total assigned
B) Including Butte Cr. and Mill/Deer creek reporting groups						
Assigned at 0.80 probability						
Fall (32)	0.91	0.09	0	0	0	84
F‐FRH (19)	0.74	0.11	0.11	0.05	0	63
L_COL (26)	0.23	0.77	0	0	0	65
L_USR (28)	0.25	0.75	0	0	0	80
S_MIL (31)	0.07	0	0.77	0.16	0	84
S_DER (30)	0.03	0	0.77	0.20	0	86
S_BUT (32)	0	0	0.03	0.94	0.03	86
S_FRH (22)	0.73	0.09	0.18	0	0	79
Winter (40)	0	0	0	0	1.00	100
Assigned at 0.60 probability						
Fall (34)	0.85	0.15	0	0	0	89
F‐FRH (27)	0.81	0.07	0.07	0.04	0	90
L_COL (33)	0.27	0.73	0	0	0	82
L_USR (34)	0.26	0.74	0	0	0	97
S_MIL (34)	0.06	0	0.74	0.21	0	92
S_DER (35)	0.03	0	0.80	0.17	0	100
S_BUT (36)	0	0	0.06	0.92	0.03	97
S_FRH (26)	0.73	0.08	0.19	0	0	93
Winter (40)	0	0	0	0	1.00	100

We were able to assign a higher proportion of samples when we used an assignment probability threshold of 0.60 compared to 0.80 (94% and 84%, respectively, when using Run as the reporting group, Table 3). Assignment accuracies were generally higher when using the more stringent 0.80 probability of assignment requirement, with the difference in accuracies between the 0.80 and 0.60 thresholds ranging from −0.07 to 0.06.

The realistic fishery simulation showed the AIM panel accurately estimates mixture proportions, with the estimated proportion being within 0.0089 on average of the simulated proportion (range: 0.0003–0.0212, Table 2).

## 4 Discussion

Here, we show how combined use of next‐generation sequencing data and targeted marker development can be used to distinguish individuals as they mix throughout their migratory pathway. We discovered thousands of new SNP markers for the culturally, economically, and ecologically important migratory Chinook salmon in the Central Valley of California. We used these new genetic resources to identify a set of SNPs that can accurately assign individuals back to their run of origin and distinct populations within Spring run. We developed these SNPs into genetic assays that have the great benefit of providing quick and easy genotypes.

Our new panel represents the highest accuracies to assign Central Valley Chinook salmon to run and population of origin thus far for any SNP panel. The previously available SNP panel obtained very low assignment accuracies within Spring (26%–68% accuracy) and Late‐fall runs (54% accuracy) (Clemento et al., 2014). Therefore, use of the previous SNP panel necessitated grouping Fall and Late fall into a single reporting group and combining all Spring run populations (Butte, Deer, and Mill creeks) into a generic Spring run reporting group in order to achieve acceptable assignment accuracies (Clemento et al., 2014). Our new panel is now able to distinguish between Fall and Late fall, as well as between populations within Spring run.

The use of this panel will greatly improve our ability to tailor management of each unique lineage of Central Valley Chinook salmon. Effective management necessitates ensuring suitable available habitat to support the different runs and populations, accurate assessment of anthropogenic impacts on each run in a timely manner, and improved tracking of habitat use and fisheries capture. Fall run is by far the most abundant run in the Central Valley and grouping it with Late fall, which has substantially smaller abundances and a much more restricted range, as has been carried out to date, risks missing declines in Late‐fall abundance and precludes evaluating actions aimed at increasing abundances. The ability to identify the Spring run individuals from the different populations will also be very important for assessing and managing the health of individual populations within this imperiled ESU. Spring run in the Central Valley were historically the most dominant run, including at least 18 independent populations (Lindley et al., 2006; Williams, 2006). Now, most of these populations have been extirpated and there are only the three watersheds supporting continuous, yet small, populations of Spring run (Lindley et al., 2007). Protecting, recovering, and possibly expanding these remaining populations requires proper management of distinct within‐run genetic resources to preserve the portfolio of Central Valley Spring run (Carlson & Satterthwaite, 2011). Our SNP panel also allows users to fine‐tune the parameters they set for assignment (i.e., different required assignment probability cutoffs for assignment). Given the small observed difference in assignment accuracies when using 0.80 and 0.60 assignment probability cutoffs, it may be desirable in some studies to use the lower threshold when the trade‐off between assignment accuracy and numbers assigned favors assigning as many individuals as possible. Additionally, practitioners will want to consider the sources of error and misallocation in any application (Seeb et al., 2007).

Understanding the effects of water management on individuals from unique Chinook salmon lineages and localities is of primary importance for species management. This includes more accurate monitoring of juvenile Chinook salmon mortality by entrainment in California's extensive state and federal water pumping operations. This water movement and export is closely managed, and rates of export are determined in part by the level of impact to listed species, including Chinook salmon runs (NMFS, 2009). Gaining a clearer understanding of exactly which Chinook salmon runs and populations are negatively impacted is paramount to effectively balancing the needs of water users with negative impacts on salmon populations.

This new SNP panel also provides the tools necessary to conduct ecological studies for each run and distinct populations of imperiled Spring run throughout their migratory pathways, as well as monitor the effects of climate change. This includes the ability to analyze diet and habitat use of each run and population independently, accurately estimate abundances, evaluate movement patterns in both freshwater and ocean environments, and conduct accurate genomewide association studies (GWAS) to determine genetic variants associated with traits of interest. Understanding these different components of the ecology of Chinook salmon in the Central Valley will be vital to predicting and planning for the effects of climate change. Previous work shows that a significant percentage (5.8%–21.8%) of the genetic variation found in Chinook salmon can be attributed to adaptive divergence driven by environmental features (Hecht, Matala, Hess, & Narum, 2015). Climate change is projected to change thermal regimes of Central Valley rivers, reduce available spawning and rearing habitat, and vary in the level of effects on the different Chinook salmon populations (Lindley et al., 2007; Thompson et al., 2012; Yates et al., 2008). Therefore, this new SNP panel will allow researchers to fine‐tune our ecological understanding of the adaptive differences among the unique populations of Chinook salmon in the Central Valley and target restoration activities to aid those populations that are most imperiled by the effects of climate change.

## 5 Conclusions

Conservation and management of threatened migratory species has been greatly limited by an inability to distinguish individuals from distinct populations as they mix along the migratory corridor. Management plans for migratory fish species that account for genetic population structure can decrease the probability of overfishing vulnerable populations and result in increased fishery catches due to the ability to manage for the optimal demographic structure (i.e., number of breeders) per population (Spies & Punt, 2015). Managing populations based on genetic distinction can also increase species resilience as well as stabilize ecosystem productivity (Hilborn et al., 2003; Schindler et al., 2010). Therefore, it is important that we have genetic tools that allow us to differentiate individuals based on the population structure present in the system.

This study demonstrates how useful genomic data can be for the management and conservation of organisms, and particularly for migratory species where distinct genetic lineages mix along migratory pathways.

Data for this study are available at the Dryad Digital Repository: http://dx.doi.org/10.5061/dryad.j29f5.

## Conflict of Interest

None declared.
